# Nurses’ Attitudes and Clinical Judgment on Skin Disinfection Before Subcutaneous Injection: Impact of Setting, Experience, and Normative Beliefs

**DOI:** 10.3390/nursrep15110393

**Published:** 2025-11-07

**Authors:** Yuko Yoshida, Kohei Ikeno, Risa Takashima

**Affiliations:** 1 Department of Nursing, Sapporo University of Health Sciences, Sapporo 007-0894, Japan; ikeno@sapporo-hokeniryou-u.ac.jp; 2 Department of Nursing Science, Graduate School of Health Sciences, Hirosaki University, Hirosaki 036-8203, Japan; 3 Department of Rehabilitation Science, Faculty of Health Sciences, Hokkaido University, Sapporo 060-0812, Japan; risa-t@hs.hokudai.ac.jp

**Keywords:** disinfection, norm, nurse, subcutaneous injection

## Abstract

**Background/Objectives**: Skin disinfection before insulin administration is widely regarded as essential for preventing injection-site infection. However, the World Health Organization advises that while hand hygiene and washing with soap and water are crucial, alcohol-based disinfection before subcutaneous injection is not required. Thus, the necessity for pre-injection (subcutaneous) skin preparation remains controversial. Therefore, this study aimed to clarify the determinants of nurses’ attitudes toward the necessity of skin disinfection before subcutaneous injection. We simultaneously examined the effects of workplace setting, years of professional experience, and social norms to identify the most significant factors influencing clinical judgment. **Methods**: Nurses employed in wards, outpatient settings, and home care settings were surveyed between October 2021 and January 2022 in this cross-sectional study. A structured questionnaire assessed frequency of skin disinfection and attitudes regarding its necessity before subcutaneous injection. Ordinal logistic regression was performed to identify factors associated with the attitude of nurses. **Results**: Overall, 992 valid responses were analyzed. Ordinal logistic regression indicated that the attitude of nurses were significantly influenced by years of professional experience (Odds Ratio [OR] = 0.98, 95% Confidence Interval [CI] [0.96, 0.99]), normative expectations (OR = 2.88, 95% CI [2.32, 3.56]), and sanctions (OR = 1.36, 95% CI [1.15, 1.62]) (all *p* < 0.001). **Conclusions**: Nurses’ beliefs regarding skin disinfection before subcutaneous injections are primarily influenced by normative expectations and professional experience, rather than workplace environment and experiential expectations. Experienced nurses do not disregard norms: they practice greater critical and situational judgment and show understanding of the purpose of disinfection.

## 1. Introduction

Skin disinfection before injection is a standard preventive measure against injection-site infections, with risks varying according to injection type and patient susceptibility [1]. However, its necessity for subcutaneous injections remains debated. The World Health Organization (WHO) does not mandate alcohol-based skin disinfection for subcutaneous injections, recommending only hand hygiene and skin cleansing with soap and water [2].

The Public Health Agency of Canada [3] advises the practice of treating skin with a suitable antiseptic solution prior to vaccination or injection. In contrast, the Forum for Injection Technique UK [4], the Australian Department of Health [5], and the Forum for Injection Technique and Therapy Expert Recommendations, India [6], state that skin disinfection before subcutaneous injection of insulin or vaccines is not necessary. Typically, patients who self-administer insulin are not instructed to disinfect before subcutaneous injection [7]. Notably, incidences of cellulitis or other local skin infections have not been reported, even in the absence of skin disinfection [8]. Several studies [9,10] have reported that nurses working in hospitals consistently perform skin disinfection before administering subcutaneous injections.

This discrepancy between evidence-based guidelines and established local practices prompted an earlier study by Yoshida et al. [11]. Their qualitative research on infection control nurses identified institutional norms as a critical determinant of this practice. The study revealed a sharp contrast in that, while disinfection is a non-negotiable protocol in hospitals, this norm is less rigid in home care settings, where patient non-adherence is often condoned. These findings suggest that the attitudes of nurses and practices regarding skin disinfection are strongly context dependent. Therefore, we hypothesized that significant differences would exist among hospital ward nurses (who adhere to strict protocols), outpatient nurses (who emphasize patient education for daily living), and home care nurses (who operate within more flexible environments).

To analyze these contextual influences, we applied social norm theory. Social norms prescribe what actions we should take or avoid, and they permeate many aspects of our behavior [12]. A social norm is a behavioral rule to which individuals conditionally conform, encompassing a personal normative belief that one ought to conform, the empirical expectation that most people in a relevant network conform, and the normative expectation that most people in the relevant network believe that one ought to conform, with deviation from the norm, may incur potential sanctions [13]. Norms have been distinguished as either behavioral regularities within a community or reference group or as shared beliefs that carry moral obligation [14]. While often studied in broad cultural contexts [15,16,17], norms have also been explored as specific determinants influencing the behavior of healthcare professionals, including nurses [18].

Beyond social-contextual factors, individual attributes are also powerful determinants of clinical practice. In particular, professional experience is widely recognized as a cornerstone of nursing expertise, influencing the development of clinical judgments that guide effective actions [19,20].

Therefore, this study aimed to clarify the determinants of nurses’ attitudes toward the necessity of skin disinfection before subcutaneous injection. We simultaneously examined the effects of workplace setting, years of professional experience, and social norms to identify the most significant factors influencing clinical judgment. Specifically, the objectives of this study were as follows:

1. Investigate whether different healthcare settings influence nurses’ attitudes, practices, and professional norms concerning skin disinfection prior to subcutaneous injections.
2. Examine the influence of professional experience on nurses’ attitudes regarding skin disinfection before subcutaneous injection.
3. Identify the specific factors (e.g., workplace setting, professional experience, and normative beliefs) that most significantly impact nurses’ attitudes regarding skin disinfection before subcutaneous injection.

## 2. Materials and Methods

### 2.1. Study Design

This study employed an observational cross-sectional design. The STROBE checklist, which is the standardized criteria for reporting cross-sectional studies, was followed [21].

### 2.2. Participants

The data were collected between October 2021 and January 2022. The target facilities included 587 home healthcare nursing agencies in Hokkaido registered on the Ministry of Health, Labor, and Welfare website, and 344 hospitals with ≥100 beds. Nurses with at least one year of clinical experience at the facilities that complied with participation were eligible. Five questionnaires were distributed to each visiting nursing office, and ten to each hospital (five for the inpatient ward and five for the outpatient department).

Facility administrators were instructed to select nurses with more experience from different departments if the pool of potential participants was large. The survey was conducted using paper-based questionnaires. Questionnaires were initially sent to facility administrators, who then distributed them to eligible nursing staff. Nurses who received the questionnaire were explicitly instructed to return their completed surveys directly to the researchers via postal mail in a pre-paid, self-addressed envelope. This method ensured that the facility administrators had no knowledge of whether individual nurses participated in the study or not, thereby guaranteeing the nurses’ ability to freely participate and ensuring confidentiality of their responses. This approach mitigated concerns that nurses might respond in ways they believed administrators would prefer. A total of 218 facilities participated, comprising 92 hospitals and 126 visiting nursing stations. A total of 1540 questionnaires were distributed: 460 to ward nurses, 450 to outpatient nurses, and 630 to home-visit nurses. Among these, 1032 questionnaires (response rate: 68.2%) and 992 valid responses were obtained.

### 2.3. Questionnaire Development

A self-administered questionnaire was designed to be completed in approximately 5–10 min, based on previous studies [11], investigating nurses’ perceptions of skin disinfection before subcutaneous injection, and researcher discussions. Content validity was assessed through consultation with a visiting nurse and a researcher and refined through a pilot study with three practicing nurses from hospital and home settings. Additionally, multiple pilot tests were conducted to ensure that the questionnaire captured both the attitudes of the nurses toward skin disinfection and its implementation (Supplementary Material).

### 2.4. Demographic Characteristics

Data on the following demographic characteristics were collected: age, years of nursing experience, education, and workplace settings (ward, outpatient, or home care).

### 2.5. Practice and Awareness of Subcutaneous Injection and Disinfection

Three categories of items were included: the frequency of administering subcutaneous injections (at least twice weekly, once weekly, once monthly, approximately once every six months, approximately annually, or not at all), frequency of skin disinfection before subcutaneous injections (scored using a five-point Likert scale ranging from 1 [not at all] to 5 [always]), and whether they had encountered patients who did not disinfect the skin before subcutaneous injections. In these cases, open-ended responses were requested to describe the patients and contexts in which disinfection was omitted. These are based on the nurses’ perceptions.

### 2.6. Norms Regarding the Practice of Skin Disinfection Before Subcutaneous Injection

Existing norms in the groups to which the nurses belonged, such as public and hospital norms, influenced the practice of performing skin disinfection before subcutaneous injections. Such practices, shaped by collective influence, are defined as social norms [13]. To assess these, questions designed by Bicchieri [13] to diagnose, explain, and predict collective behavioral patterns were used. Items assessing empirical expectations, normative expectations, personal normative beliefs, and sanctions were adapted from previous studies [18,22]. Specifically: personal normative beliefs were assessed with the item, “Skin disinfection should always be performed before subcutaneous injection”; normative expectations were assessed with, “Most nurses around you think that skin disinfection should be performed before subcutaneous injection”; empirical expectations were assessed with, “Most nurses around you actually perform skin disinfection before subcutaneous injection”; and sanctions (a component of normative expectations) were assessed with the item, “If you did not perform skin disinfection, most nurses around you would probably notice and reprimand you”. Responses were recorded on a six-point scale (very much agree, agree, somewhat agree, not much agree, disagree, or not at all agree). The 4-item scale for norms showed adequate internal consistency (Cronbach’s alpha = 0.71).

### 2.7. Statistical Analysis

Data were analyzed using IBM SPSS Statistics for Windows, version 30.0 (IBM Corp., Armonk, NY, USA). Age, nursing experience, education, frequency of subcutaneous injection practice, and frequency of disinfection before subcutaneous injection were reported as mean ± standard deviation or number (percentage), as appropriate. Statistical significance was set at *p* < 0.05. Age and nursing experience were analyzed using ANOVA and Tukey’s HSD test was then applied for pairwise comparisons. The latter three were analyzed using Pearson’s chi-square test. Norms regarding disinfection practices before subcutaneous injection were analyzed using Kruskal–Wallis test. Post hoc analyses were performed using Mann–Whitney U tests with Bonferroni correction. Ordinal logistic regression analysis was conducted to identify the factors influencing nurses’ attitudes toward disinfection. The dependent variable was measured using a 6-point ordinal Likert scale. The dependent variable was in agreement with the statement: “Skin disinfection should always be performed before subcutaneous injection.” Based on the theoretical framework and relevant literature, the independent variables included workplace setting (hospital ward, outpatient settings, and home care setting), years of professional experience (as a continuous variable), and three measures of social norms: normative expectations, empirical expectations, and sanctions. For the workplace setting, “Hospital Ward” was set as the reference category. All the independent variables were entered simultaneously (entry method). The results are presented as odds ratios (OR) with corresponding 95% confidence intervals (CI). Sensitivity analyses were performed to evaluate the robustness of the findings. First, the test of parallel lines in the ordinal regression model indicated a violation of the proportional odds assumption for certain variables (*p* < 0.05). As an alternative, the dependent variable was dichotomized at the median, and binary logistic regression was performed. The results from this binary logistic regression were largely consistent with the primary ordinal regression regarding the direction and significance of the effects of the key independent variables. Second, Cook’s distance was calculated using a preliminary linear regression model to assess the influence of potential outliers. All observations were retained in the primary analysis, as no single case was found to exert an undue influence on the model results. The proportional odds assumption for the model was verified using parallel line tests. For ordinal logistic regression analysis, listwise deletion was employed to handle missing data, ensuring that all cases included in the regression models had complete data across all variables. Responses from questionnaires that met these criteria were considered valid responses. No imputation methods were used for the descriptive statistics. Therefore, in the dataset, ‘Education’ had missing data for three participants, and ‘whether skin disinfection was performed before subcutaneous injection’ had missing data for one participant. A total of 992 valid responses were obtained.

### 2.8. Qualitative Analysis

Open-ended responses were analyzed using qualitative content analysis [23]. The first author read all responses and coded the data, grouping similar responses into descriptive categories. The categories were then reviewed and discussed among all co-authors until consensus was reached to ensure trustworthiness and confirmability [24].

### 2.9. Ethics

This study adhered to the tenets of the Declaration of Helsinki. The study protocol was approved by the Ethics Committee of Sapporo University of Health Sciences (021009-3). All participants voluntarily participated in this study. Written information about the study was provided before participation, and informed consent was obtained through voluntary completion and submission of an anonymous questionnaire. The participants were also assured of their confidentiality and anonymity.

## 3. Results

Table 1 presents the participants’ characteristics. A total of 992 participants (mean age: 47.3 ± 9.5 years; and mean years of nursing experience: 23.2 ± 9.8 years) were included in this study. Skin disinfection was performed before subcutaneous injection in 99.6% of the cases. Kruskal–Wallis tests showed that all four social norm scores differed significantly across the three workplace settings (*p* < 0.05). In post hoc tests, home care nurses reported significantly lower scores for normative expectations, empirical expectations, and sanctions than both hospital ward nurses and outpatient nurses. For personal normative beliefs, their scores were significantly lower only when compared to those of hospital ward nurses. There were no significant differences in any of the norm variables between hospital ward nurses and outpatient nurses. Notably, 259 participants (26.1%) had encountered patients who did not perform skin disinfection before subcutaneous injections (Table 2). As shown in Table 2, the most frequently reported reason was habituation (*n* = 92, 29.5%), followed by cognitive impairment (*n* = 70, 22.4%).

Table 3 demonstrates the findings of the ordinal logistic regression analysis examining factors affecting the attitude of nurses on skin disinfection. Professional experience, normative expectations, and sanctions were significant predictors. The results of the analyses showed that nurses’ attitudes were strongly influenced by their years of professional experience (OR = 0.98, 95% CI [0.96, 0.99], *p* < 0.001), the normative expectations (OR = 2.88, 95% CI [2.32, 3.56], *p* < 0.001) and sanctions (OR = 1.36, 95% CI [1.15, 1.62], *p* < 0.001). In contrast, workplace setting (outpatient settings or home care setting compared to hospital ward) and empirical expectations were not significantly associated with attitudes toward skin disinfection (*p* > 0.05).

## 4. Discussion

This study revealed that the most significant factors determining the attitude of nurses toward skin antisepsis before subcutaneous injections were normative expectations (what others think should be done) and years of experience, rather than the environment of the workplace. Interestingly, the workplace and experiential expectations (what others actually do) had no direct influence.

The negative association between years of experience and implementation of skin antisepsis before subcutaneous injections does not suggest that experienced nurses disregard norms. With experience, nurses gain a deeper understanding of the true purpose of antisepsis (infection prevention) and, in line with the WHO guidelines [2], acquire the ability to make more critical, context-sensitive clinical judgments, recognizing that antisepsis is not uniformly necessary in all situations. This may be seen as a maturation from rule-following practice to making judgments based on evidence and situation. This finding is further supported by previous studies indicating that clinical judgment ability is higher among nurses with greater autonomy [25,26]. This maturation from rule-following practice to making judgments also based on evidence and situation aligns well with Patricia Benner’s “From Novice to Expert” theory [27].

Drawing on findings from a previous study [11], this study hypothesized that nurses working in home care settings often encounter situations where patients and their families administer subcutaneous injections, and therefore may not believe that strict adherence to aseptic techniques is necessary in hospitals. Therefore, we hypothesized that home care nurses may not adhere to aseptic techniques as in hospitals. However, the results of this study suggest that home care nurses provide care tailored to each patient based on extensive clinical experience rather than on the presence or absence of norms.

Furthermore, the lack of significance in “experiential expectations” suggests that the judgment of nurses are not driven by simple behavioral imitation such as “everyone else is doing it,” but rather by strong adherence to the beliefs of their professional group. This is an important finding of this study. The significance of “normative expectations” suggests that these professional standards influence decisions regarding skin disinfection before subcutaneous injections. This finding is consistent with research in other clinical contexts that has also highlighted the influence of social norms on healthcare professionals’ decision-making. For instance, physicians prescribing antibiotics have reported that pressure from patients and other physicians influences their prescribing behavior [28]. Previous research on clinical judgment has often emphasized individual factors, such as knowledge or competency [19,20]. However, our findings indicate that this judgment is not shaped by personal knowledge and experience alone; instead, it is strongly influenced by normative expectations within the professional team or organization.

Table 2 demonstrates that qualitative data, showing that “cognitive impairment” was the common reason for omitting disinfection, highlighting the complexity of the clinical environment that nurses face. Although nurses believe that “disinfection should be done” as a rule, in reality, they encounter many situations where this is difficult due to patient-related factors. However, omissions of skin disinfection before subcutaneous injections may also be tolerated, as they believe that forgetting to do so is unlikely to result in a fatal situation [8,29]. Given that healthcare settings extend beyond hospitals, for patients undergoing self-injection of insulin, for example, there is also a recognized need to update best practices for injection procedures, stratified by each specific clinical setting [30].

Performing skin disinfection before injection is deeply ingrained in patients’ minds; thus, some individuals consider it common sense to perform skin disinfection before all injections. Therefore, de-implementing certain care and intervention practices that are currently being implemented in clinical practice is difficult [31]. De-implementation is defined as discontinuing practices that are not evidence-based [32]. Low-value care refers to care that is unlikely to benefit the patient given the harm, cost, available alternatives, or patient preferences [33]. Depending on the patient’s condition or environmental circumstances, skin disinfection performed by the patient before subcutaneous injections administered in the home setting may also belong to this category. De-implementation of practices such as skin disinfection before subcutaneous injections is challenging because it is unlikely to be achieved solely through guideline revisions or unilateral information provision to nurses and patients. Cross-cultural comparison research reveals the idiosyncratic nature of social norms, demonstrating how actions considered appropriate in one culture can be deemed highly deviant in another [34,35]. This study suggests that clinically experienced nurses make judgments based on a comprehensive consideration of both established protocols and individual patient needs. Promoting the de-implementation of practices that may change depends on the content could find a potential solution in cross-cultural research.

For future nursing education, providing opportunities within educational curricula to understand how normative expectations within a professional group are formed and influence clinical judgments is expected to be beneficial. This perspective could be effectively integrated as part of clinical judgment education and professional ethics education. Additionally, providing opportunities during education and training for reflection on the background of clinical decisions (including purposes, evidence, and their relationship with organizational norms) by asking questions such as, “Why do we do this?” may be helpful. This approach can help nurses move beyond formal compliance and foster the ability to make evidence-based and context-sensitive judgments.

Furthermore, while guidelines and forums serve as critical references for professional judgment and influence norm formation, currently, a complex situation prevails where various guidelines and forums present conflicting information regarding “best practice.” It is therefore imperative that international discussions are held to resolve this controversy and establish a consolidated best practice.

This study has serval limitations. First, as it focused solely on nurses working in Japan, the findings may not be generalizable to other environments. Second, while content validity and internal consistency were assessed, comprehensive validation, such as factor analysis and criterion validity, was not performed. Third, the response rate of 68.2% raises the possibility of a selection bias. However, considering the general response rate of mail surveys [36], the data can be considered sufficiently practical and meaningful. Fourth, in this study, we indeed intentionally focused on nurses with more extensive clinical experience, while deliberately avoiding newer nurses, to gather insights from seasoned professionals. Consequently, the findings of this study may not be generalizable to novice nurses with limited clinical experience. Fifth, despite efforts to ensure anonymity and voluntary participation through direct mailing, selection bias remains a potential concern. Facility administrators, who distributed the questionnaires, might have selected nurses based on their perceived attitudes toward skin disinfection, influenced by their own beliefs. This potential bias could affect sample representativeness and result interpretation. Furthermore, the cross-sectional design of this study limits its ability to draw causal inferences.

## 5. Conclusions

This study revealed that the belief of nurses in always performing skin disinfection before subcutaneous injections is influenced by normative expectations rather than empirical expectations. This relationship was consistent across workplace settings but varied in years of professional experience. These findings suggest that norms held by nursing professionals are key determinants in shaping clinical practice. Future research that compares nurses across different cultural contexts may reveal the extent to which cultural norms permeate the nursing profession.

## Figures and Tables

**Table 1:** Summary of participant characteristics and univariate analysis results.

Variables	All (*n* = 992)	Ward Nurse (*n* = 323)	Outpatient Nurse (*n* = 328)	Visiting Home Health Nurse (*n* = 341)	*p*
Age, years	47.3 ± 9.5	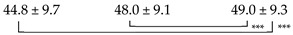			<0.001 ***
		
Nursing experience, years	23.2 ± 9.8	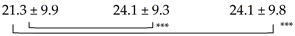			<0.001 ***
Education					0.020 ***
Professional training college	822 (83.1%)	282 (87.3%)	274 (83.8%)	266 (78.5%)	
Junior college	55 (5.6%)	12 (3.7%)	18 (5.5%)	25 (7.4%)	
Bachelor	65 (6.6%)	17 (5.3%)	16 (4.9%)	32 (9.4%)	
Master or above	11 (1.1%)	2 (0.6%)	1 (0.3%)	8 (2.4%)	
No answer	36 (3.6%)	10 (3.1%)	18 (5.5%)	8 (2.4%)	
Frequency of subcutaneous injection practice					<0.001 ***
More than twice a week	348 (35.1%)	116 (35.9%)	177 (54.0%)	55 (16.1%)	
More than once a week	244 (24.6%)	91 (28.2%)	54 (16.5%)	99 (29.0%)	
More than once a month	164 (16.5%)	53 (16.4%)	43 (13.1%)	68 (19.9%)	
More than half a year	103 (10.4%)	39 (12.1%)	19 (5.8%)	45 (13.2%)	
About once a year	74 (7.5%)	19 (5.9%)	22 (6.7%)	33 (9.7%)	
None	59 (5.9%)	5 (1.5%)	13 (4.0%)	41 (12.0%)	
Whether skin disinfection is subcutaneous injection performed before					0.408
Always	987 (99.6%)	321 (99.4%)	328 (100%)	338 (99.4%)	
Often	1 (0.1%)	0 (0%)	0 (0%)	1 (0.3%)	
Sometimes	0 (0%)	0 (0%)	0 (0%)	0 (0%)	
Almost never	3 (0.3%)	2 (0.6%)	0 (0%)	1 (0.3%)	
Not at all	0 (0%)	0 (0%)	0 (0%)	0 (0%)	
Norms					
Personal normative beliefs	5.0 ± 1.0	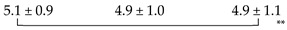			0.045 *
Normative expectations	5.3 ± 0.7	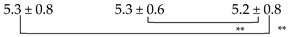			0.045 *
Empirical expectations	5.6 ± 0.5	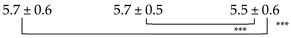			<0.001 ***
Sanctions (normative expectations)	5.3 ± 0.9	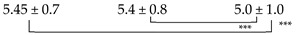			<0.001 ***

Note. Statistical significance = *** *p* < 0.001; ** *p* < 0.01; * *p* < 0.05. ANOVA: Age, Nursing experience. Pearson’s chi-squared tests: Education, Frequency of subcutaneous injections practice, Whether skin disinfection is performed before subcutaneous injection. Kruskal–Wallis test: norms. Missing data: Education (*n* = 3) and skin disinfection before subcutaneous injection (*n* = 1).

**Table 2:** Nurses’ perceptions of reasons and contexts for patients omitting skin disinfection.

Category	Description of Category	*n*	%
1. Habituation/Self-Styled Routine	Skipping steps due to over-familiarity with the procedure from long-term self-injection, or establishing a personal, non-standard routine.	92	29.5
2. Cognitive Impairment	Forgetting or being unable to understand the disinfection procedure due to dementia or age-related cognitive decline.	70	22.4
3. Perceived Burden/Hassle	Omitting disinfection because the process is perceived as bothersome, troublesome, or a burden in a busy lifestyle.	50	16.0
4. Lack of Skill or Understanding	Not having yet mastered the self-injection technique or not fully understanding/accepting the necessity of disinfection.	34	10.9
5. Instructions from Other Healthcare Providers	Being previously instructed by a physician or nurse at another facility that disinfection is unnecessary.	24	7.7
6. Lack of Supplies/Physical Barriers	Inability to perform disinfection due to physical reasons, such as running out of alcohol swabs, forgetting to prepare them, cost issues, or not having them on hand when outside the home.	18	5.8
7. Specific Situations/Environments	Omitting disinfection in specific non-hospital settings, such as at home, outdoors, or at the workplace.	8	2.6
8. Injecting Through Clothing	Injecting directly through clothing to avoid the hassle of exposing the skin.	6	1.9
9. Impact of Mental Illness	Difficulty performing self-care behaviors due to mental health conditions such as schizophrenia or depression.	5	1.6
10. Skin Problems/Allergies	Avoiding disinfection to prevent skin irritation, redness, or allergic reactions caused by alcohol swabs.	5	1.6
Total		312	100

Note. The data were derived from open-ended responses from 259 nurses who reported having encountered patients who omitted skin disinfection. The total number of cases (*n* = 312) exceeds the number of nurses because some nurses reported multiple cases. Percentages may not sum to 100.0 due to rounding.

**Table 3:** Ordinal logistic regression analysis predicting nurses’ attitudes toward skin disinfection before subcutaneous injection.

						CI	
Variable	B	SE	Wald	*p*-Value	OR	Lower	Upper
Clinical Setting							
Workplace (Reference: Hospital Ward)							
Outpatient Settings	−0.21	0.15	1.91	0.168	0.81	0.60	1.090
Home Care Setting	−0.09	0.15	0.32	0.574	0.92	0.68	1.24
Professional Experience							
Years of Experience	−0.02	0.01	14.23	<0.001	0.98	0.96	0.99
Normative Beliefs							
Normative Expectations	1.06	0.11	93.45	<0.001	2.88	2.32	3.56
Empirical Expectations	−0.12	0.15	0.7	0.403	0.89	0.67	1.18
Normative Expectations (Sanctions)	0.31	0.09	12.63	<0.001	1.36	1.15	1.62

Note. The dependent variable was the level of agreement with skin disinfection that should always be performed before subcutaneous injection (6-point Likert scale). The overall model was statistically significant (χ^2^(6) = 229.965, *p* < 0.001. The deviance goodness-of-fit test indicated that the model fit the data well (χ^2^(2689) = 1482.364, *p* = 1.000), and Nagelkerke’s R^2^ was 0.224. B, unstandardized coefficient; SE, standard error; Wald, Wald chi-square value; OR, odds ratio; CI, confidence interval.

## Data Availability

All data generated or analyzed during this study are available from the corresponding author upon reasonable request. The data are not publicly available due to privacy and ethical restrictions.

## Supplementary Materials

The following supporting information can be downloaded at: https://www.mdpi.com/article/10.3390/nursrep15110393/s1, Supplementary Material 1: The Questionnaire used in this study.

## Public Involvement Statement

There was no public involvement in any aspect of this research.

## Guidelines and Standards Statement

This manuscript was drafted against the STROBE (Strengthening the Reporting of Observational Studies in Epidemiology) for cross-sectional studies. A complete list of reporting guidelines can be accessed via the equator network: https://www.equator-network.org/, accessed on 28 March 2025.

## Abbreviations

The following abbreviations are used in this manuscript:

WHO	World Health Organization
OR	Odds Ratio
CI	Confidence Interval
