# Supplementary material for: Nurses’ Attitudes and Clinical Judgment on Skin Disinfection Before Subcutaneous Injection: Impact of Setting, Experience, and Normative Beliefs
Source: Nurs Rep. 2025 Nov 7;15(11):393. doi: 10.3390/nursrep15110393 (PMC12655181; doi:10.3390/nursrep15110393)
Supplement: Supplementary file 1 [file nursrep-15-00393-s001.zip › nursrep-3876357-supplementary.pdf]

Ⅰ. Your usual practice regarding insulin injection techniques

Q1. How often do you administer subcutaneous injections? (Please circle one)

1. Twice a week or more
2. About once a week
3. About once a month
4. About once every six months
5. About once a year
6. Never

Q2. How often do you administer skin disinfection before subcutaneous infections?

(Please circle one)

1. Not at all
2. Almost never
3. Sometimes
4. Often
5. Always

Q3. Have you ever encountered someone who did not disinfect the skin before a subcutaneous injection? (Please circle one)

1. Yes
2. No

Q4. For those who answered "Yes" to Q3, please tell us: In what specific situations or with what kind of patients did this occur? Please describe.

Ⅱ. Please circle the answer that best reflects your opinion.

Q1. Skin disinfection should always be performed before subcutaneous injection.

1. Not at all agree
2. Disagree
3. Not much agree
4. Somewhat agree
5. Agree
6. Very much agree

Q2. Most nurses around you think that skin disinfection should be performed before subcutaneous injections.

1. Not at all agree
2. Disagree
3. Not much agree
4. Somewhat agree
5. Agree
6. Very much agree

Q3. Most nurses around you actually perform skin disinfection before subcutaneous injections.

1. Not at all agree
2. Disagree
3. Not much agree
4. Somewhat agree
5. Agree
6. Very much agree

Q4. If you did not perform skin disinfection, most nurses around you would probably notice and reprimand you.

1. Not at all agree
2. Disagree
3. Not much agree
4. Somewhat agree
5. Agree
6. Very much agree

Ⅲ. About Yourself

Q1. Age

Q2. Years of nursing experience

Q3. Highest level of education (Please circle one)

1．Vocational school
2．Junior college/Associate degree
3．University/Bachelor’s degree
4．Graduate school (Master’s program)/Master’s degree
5．Graduate school (Doctoral program)/Doctoral degree
